# Supplementary material for: Dietary diversity and evolution of the earliest flying vertebrates revealed by dental microwear texture analysis
Source: Nat Commun. 2020 Oct 28;11:5293. doi: 10.1038/s41467-020-19022-2 (PMC7595196; doi:10.1038/s41467-020-19022-2)
Supplement: Supplementary file 1 — Supplementary Information [file 41467_2020_19022_MOESM1_ESM.pdf]

Supplementary Information for:

Dietary diversity and evolution of the earliest flying vertebrates revealed by dental microwear texture analysis

Bestwick et al.

This PDF file includes:

Supplementary Notes 1–3

Supplementary Figures 1–11

Supplementary Tables 1–2

Supplementary References 1–21

Supplementary Note 1: Dental microwear textural analysis of extant bats with well-constrained dietary differences

ANOVA of non-occlusal tooth microwear textures between bat dietary guilds reveal 17 parameters significantly differ between guilds (Supplementary Data 3[1]). Tukey HSD pairwise testing of the significant parameters reveal no significant pairwise differences for Sku and Smr2. These parameters were therefore not included in the bat texture-dietary space that comprised significant texture parameters. Carnivores differ from ‘harder’ invertebrate consumers for six parameters (higher Sds, lower Vmc, Vvc, Sk and Sa); differ from ‘softest’ invertebrate consumers for 11 parameters (lower Sq, Sp, Sv, Sz, Vmp, Vmc, Vvc, Spk, Sk, S5z and Sa); but do not differ from piscivores. ‘Harder’ invertebrate consumers differ from ‘softest’ invertebrate consumers for six parameters (lower Sds, Str, Ssc, Spk, Smr1 and S5z); but do not differ from piscivores. ‘Softest’ invertebrate consumers differ from piscivores for seven parameters (higher Sp, Sz, Vmp, Vvc, Spk, Smr1 and S5z).

Further exploratory analysis reveals more textural differences associated with diet. Matched Pairs t-tests (Supplementary Tables 1–2) reveal that ‘softest’ invertebrate consumers differ from all guilds and ‘harder’ invertebrate consumers differ from piscivores ($P < 0.05$). The ‘harder’ invertebrate consumers and ‘softer’ invertebrate consumers have the highest average values (1st rank) for 20 of 21 parameters, including 14 of the 15 parameters that comprise the texture-dietary space. In simple terms, the surface textures of invertebrate consuming guilds are rougher than textures of vertebrate consuming guilds (Supplementary Fig. 3a–d). These results are broadly similar to microwear texture differences between vertebrate and invertebrate consuming guilds of extant reptiles[2, 3]. Reasons for why they are not the same are unclear but may reflect different tooth replacement patterns between reptiles and mammals. Further investigation into this, however, is beyond the scope of this study. These results not only demonstrate the power of DMTA as a technique for reconstructing diets from non-occlusal tooth surfaces, but also supports the use of unrelated taxa as independent multivariate frameworks for reconstructing diets of extinct reptiles such as pterosaurs.

ANOVA of the texture-dietary space results (Supplementary Fig. 3e) provide further support of microwear differences between bat dietary guilds. PC axes 1 and 2 both differ between guilds (PC 1, $F = 8.2432$, d.f. = 3, 55, $P = 0.0001$; PC 2, $F = 12.1889$, d.f. = 3, 55, $P < 0.0001$). Tukey HSD testing reveals a number of pairwise differences: across PC axis 1, 'softest' invertebrate consumers differ from carnivores and piscivores; across PC axis 2, 'harder' invertebrate consumers differ from carnivores and 'softest' invertebrate consumers, and piscivores differ from carnivores and 'softest' invertebrate consumers.

Supplementary Note 2: Projection of pterosaur microwear textures into the extant bat texture-dietary space

Projecting pterosaur data into the bat texture-dietary space reveals similar distributions of specimens and taxa as in the reptile texture-dietary space (Supplementary Fig. 3e). This high degree of consistency in evidence for diets in pterosaurs, a stringent test given the differences between these taxa, provides compelling evidence for the power of our approach, and that pterosaur microwear texture differences are attributable to dietary differences. This allows us to further test dietary hypotheses and provide more refined characterisations of pterosaur ecology. *Istiodactylus*, for example, is placed at the extreme end of the total vertebrate/carnivore scale along PC 1 (Supplementary Fig. 3e), providing independent evidence for this pterosaur as an obligate vertebrate consumer. Overlap with bat piscivores along PCs 1 and 2 indicates that fish cannot be excluded components of the *Istiodactylus* diet. However, the dietary correlations along PC 1 in the bat texture-dietary space (Supplementary Fig. 3e) interpreted together with the pterosaur's position in the reptile texture-dietary space (Fig. 2), and previous interpretations of diet based on comparative anatomy[4] indicate that *Istiodactylus* was most likely a carnivore.

Similar to the reptile texture-dietary space, *Serradraco* has values that fall within the range of piscivores (Fig. 2; Supplementary Fig. 3e), further indicating that this poorly known pterosaur was piscivorous. However, PC values also fall within the ranges of 'harder' invertebrate consumers (Supplementary Fig. 3e) suggesting that 'harder' invertebrates may have been a component of its

diet. *Lonchodraco* exhibits a slightly more positive PC 1 value than *Serradraco*, falling within the ranges of bat invertebrate consumers (for PC 2 also) and carnivores (Supplementary Fig. 3e). Combining this result with the reptile analysis indicates a diet primarily comprising vertebrates with some invertebrates.

Some pterosaurs that plot as mixed vertebrate-invertebrate consumers in the reptile texture-dietary space exhibit higher degrees of overlap with bat invertebrate consumers along PC 1 (Supplementary Fig. 3e). *Darwinopterus*, for example, has been interpreted as a carnivore, piscivore and insectivore based on descriptions of its dentition and associations with contemporaneous taxa (ref. [5] and references therein). DMTA provides the first quantitative dietary constraints for this pterosaur. The PC 1 and PC 2 values for *Darwinopterus* are most similar to bats that consume 'harder' invertebrates (Supplementary Fig. 3e). This suggests that *Darwinopterus* was an almost exclusive invertebrate consumer, and while this is consistent with its position in the reptile texture-dietary space, it also overlaps with carnivores and piscivores in the latter analysis (Fig. 2). Some consumption of vertebrates cannot be ruled out on the basis of our DMTA.

Austriadactylus and some *Pterodactylus* specimens exhibit positive PC 1 values that extend beyond the range of bat invertebrate consumers (Supplementary Fig. 3e). Rougher textures in these pterosaurs suggest consumption of invertebrates such as crustaceans and shelled molluscs, more similar to reptile 'harder' invertebrate consumers[6, 7], which is consistent with the reptile analysis (beetles are the only 'hard' invertebrates consumed by the bats studied[8]).

Turning attention to sympatric pterosaurs, *Rhamphorhynchus* and *Pterodactylus*, as they do in the reptile analysis, exhibit significant separation in their distribution with respect to PC 1 (t = -4.179, d.f. = 18, P = 0.0006), but not PC 2 (t = 1.286, d.f. = 18, P = 0.2148; Supplementary Fig. 3e). *Rhamphorhynchus* tends towards more negative PC 1 values, indicative of consumption of higher proportions of vertebrates; specimens that are likely to be adults (based on their size and degree of skeletal ossification[9]) overlap most strongly with piscivores along PCs 1 and 2 (Supplementary Fig. 3e). *Pterodactylus* individuals, in contrast to *Rhamphorhynchus* but again like the reptile analysis,

exhibit a broad range of positive PC 1 values, indicating invertebrate-dominated, generalist diets (Supplementary Fig. 3e). This provides further evidence of niche partitioning between sympatric pterosaurs from the Solnhofen archipelago and the ecological roles they performed within this ecosystem. Microwear from *Germanodactylus* and *Scaphognathus* fall within the ranges of values for bat invertebrate consumers on PCs 1 and 2, and within the range of carnivores only on PC 1 (Supplementary Fig. 3e). Interpreting the placements of these pterosaurs in both the reptile and bat texture-dietary spaces (Fig. 2; Supplementary Fig. 3e) suggests mixed vertebrate-invertebrate diets with the latter more frequently consumed. Very similar PC 1 and 2 values to some *Rhamphorhynchus* and *Pterodactylus* specimens (Supplementary Fig. 3e) again suggests potential competition between *Germanodactylus* and *Scaphognathus* with other Solnhofen pterosaurs.

Rhamphorhynchus specimens again exhibit separation along PC 1 that correlates with specimen size (lower jaw length; r_s = -0.789, P = 0.0008; Supplementary Fig 4). *Rhamphorhynchus* PC 2 values do not correlate with specimen size (r_s = -0.187, P = 0.523). The most immature individuals exhibit positive PC 1 values, indicating invertebrate-dominated diets, and late-stage juveniles and adults exhibit decreasing PC 1 values and thus increasingly piscivorous diets (Supplementary Figs. 3e, 4). Further evidence of ontogenetic niche shifts in *Rhamphorhynchus* not only indicates that pterosaurs were vital components in Mesozoic ecosystems by having multiple ecological roles within a single species, but also further supports the hypothesis that pterosaur life-histories were similar to those of extant reptiles[9–12].

Supplementary Note 3: Pterosaur dietary evolution using the Andres & Myers[13] and Wang et al.[14] phylogenies

There is a significant phylogenetic signal in pterosaur microwear along PC 1 in the Andres & Myers[13] phylogeny (K = 0.8383, P = 0.038, λ = 0.7314, P = 0.0268), and no signal along PC 2 (K = 0.2668, P = 0.623, λ = 0.0000661, P = 0.1; Supplementary Fig. 7). The ancestral PC 1 value estimate of Pterosauria (node 1, Supplementary Fig. 10) is 0.904, and the ancestral PC 2 estimate is 0.263 (Supplementary Figs. 7, 10). This provides independent evidence that the ancestral

pterosaur diet was likely invertebrate-dominated. In common with the analysis based on Lü et al.[15], pterosaurs move through phylo-texture-dietary space over time to occupy increasingly negative PC 1 values, reflecting a shift towards consumption of more vertebrates (Supplementary Fig. 7 a–b). The invertebrate-dominated diet of *Pterodactylus* is again secondarily derived (Supplementary Fig. 7 a–c). In contrast, however, the most recent common ancestor of *Istiodactylus* and *Serradraco* (Ornithocheiroidea, node 13; Supplementary Fig. 10) has a highly negative value of -2.33 (Supplementary Data 6[1]), which indicates that obligate vertebrate consumption did not evolve independently in these taxa. Also, *Lonchodraco*, *Coloborhynchus* and, to a lesser extent, *Anhanguera*, all exhibit independent shifts towards less negative PC 1 values, indicating consumption of fewer vertebrates (Supplementary Fig. 7b)

Unlike the other two analyses, pterosaur dietary microwear, as represented by PC 1, carries no phylogenetic signal in the analysis based on the Wang et al.[14] phylogeny (K = 0.5526, P = 0.074, λ = 0.9379, P = 0.0501), or along PC 2 (K = 0.2303, P = 0.833, λ <0.0001, P = 1; Supplementary Fig. 8). However, the ancestral PC 1 value estimate of Pterosauria (node 1, Supplementary Fig. 11) is 1.14 and the ancestral PC 2 estimate is 0.242 (Supplementary Fig. 8; Supplementary Data 6[1]), again providing evidence that the ancestral pterosaur diet was invertebrate-dominated. Multiple pterosaur taxa and lineages exhibit independent shifts along PC 1 to occupy increasingly negative PC 1 values, and thus consumption of more vertebrates, including (but not exclusive to); *Campylognathoides*, *Rhamphorhynchus*, *Scaphognathus, Dorygnathus* and the clade Monofenestrata (node 7, Supplementary Figs. 8, 11). Monofenestratan pterosaurs occupy increasingly negative PC 1 values over time, so much so that the most recent common ancestor of Anhangueria + *Istiodactylus* (node 14; Supplementary Fig. 11) exhibiting a value of -3.16 (Supplementary Fig 8; Supplementary Data 6[1]), indicating obligate vertebrate consumption. Multiple taxa exhibited shifts towards less negative PC 1 values, including *Anhanguera*, *Coloborhynchus*, *Lonchodraco* and, to a lesser extent, *Haopterus* (Supplementary Fig. 8). *Pterodactylus* once again shifts towards a positive PC 1 value, and thus a secondarily derived invertebrate-dominated diet (Supplementary Fig. 8a–b).

Overall, the phylogenetic mapping and ancestral state reconstructions from each of the three phylogenetic trees provides broad and independent support to the hypothesis that pterosaurs evolved as invertebrate consumers and later evolved into piscivores and carnivores[16, 17]. However, key differences between the phylogenies affect our understanding of several aspects of pterosaur dietary evolution. For example, the ancestral dietary estimate of Pterosauria in the Lü et al.[15] phylogeny has the most positive PC 1 and 2 values (albeit with broad 95% confidence intervals in each of the three phylogenies; Supplementary Data 6[1]). The ancestral pterosaur diet in this phylogeny thus likely contains the highest proportions of invertebrates. The 'harder' invertebrate-rich diet of *Austriadactylus* (Fig. 4a; Supplementary Figs. 7a, 8a) therefore represents the plesiomorphic condition in the Lü et al.[15] phylogeny and a derived condition in the Andres & Myers[13] and Wang et al.[14] phylogenies (Fig. 4b; Supplementary Figs. 7b, 8b). Moreover, the phylogenies exhibit different degrees of parsimony with respect to the numbers of independent dietary shifts exhibited by clades and/or taxa along PCs 1 and 2. The Andres & Myers[13] phylogeny could be argued to be the most parsimonious across Pterosauria as few clades and taxa repeatedly shift back and forth along PC 1 and thus exhibit few repeated dietary shifts (Supplementary Fig. 7; Supplementary Data 6[1]). The Lü et al.[15] phylogeny, however, can be considered the most parsimonious with respect to diet across Pterodactyloidea (a clade recognised in all three phylogenies) with the fewest dietary shifts away from near-obligate vertebrate consumption (Fig. 4). The Wang et al.[14] phylogeny could be argued to be the least parsimonious with respect to diet as independent shifts occur toward increasingly positive PC 1 and 2 values in several taxa and clades (examples in previous paragraph; Supplementary Fig. 8). This high number of implied dietary transitions may be due, in part, to the relative lack of resolution in the relationships between non-pterodactyloid pterosaurs in this phylogeny[14, 18, 19] which potentially explains the lack of a phylogenetic signal in the phylo-texture-dietary space (Supplementary Fig. 8). In all three phylogenies, pterosaur shifts along PC 2 are generally taxon rather than clade-specific (Supplementary Figs. 6, 7c, 8c), which indicates rapid but relatively subtle dietary shifts. While the aim of our study is not to assess the robustness of pterosaur phylogenetic trees, our results indicate that choice of phylogeny has an impact on some details of our analysis of pterosaur dietary evolution. Better phylogenetic resolution and greater

consensus regarding pterosaur relationships will lead to improved stability in evolutionary analyses, including quantitative investigations of their dietary evolution using DMTA.

Although our taxonomic sampling is a reduced subset of the total phylogeny of Pterosauria, observed differences between the three phylogenies also have marked implications for understanding the potential drivers of pterosaur dietary evolution. For example, in the Andres & Myers[13] phylogeny, pterosaurs such as *Dimorphodon* and *Campylognathoides* do not independently evolve mixed vertebrate-invertebrate diets. Rather, they share a plesiomorphic shift towards diets with a higher proportion of vertebrates with more derived clades. The dietary shift therefore began in the Upper Triassic in the Andres & Myers[13] phylogeny as opposed to the Lower–Middle Jurassic in the Lü et al.[15] and Wang et al.[14] phylogenies. Dietary shifts in the Upper Triassic may therefore be the result of ecological radiations, as opposed to potential competitive displacement by other flying taxa, as birds did not evolve until the Upper Jurassic[16, 17, 20]. However, competition with more basal lineages of theropod dinosaurs during the Upper Triassic and Lower Jurassic as a potential driver of dietary shifts cannot be ruled out[21]. Resolution of these uncertainties regarding dietary competition and co-existence between pterosaurs and birds will require better constrained hypotheses of pterosaur phylogenetic relationships and divergence times.

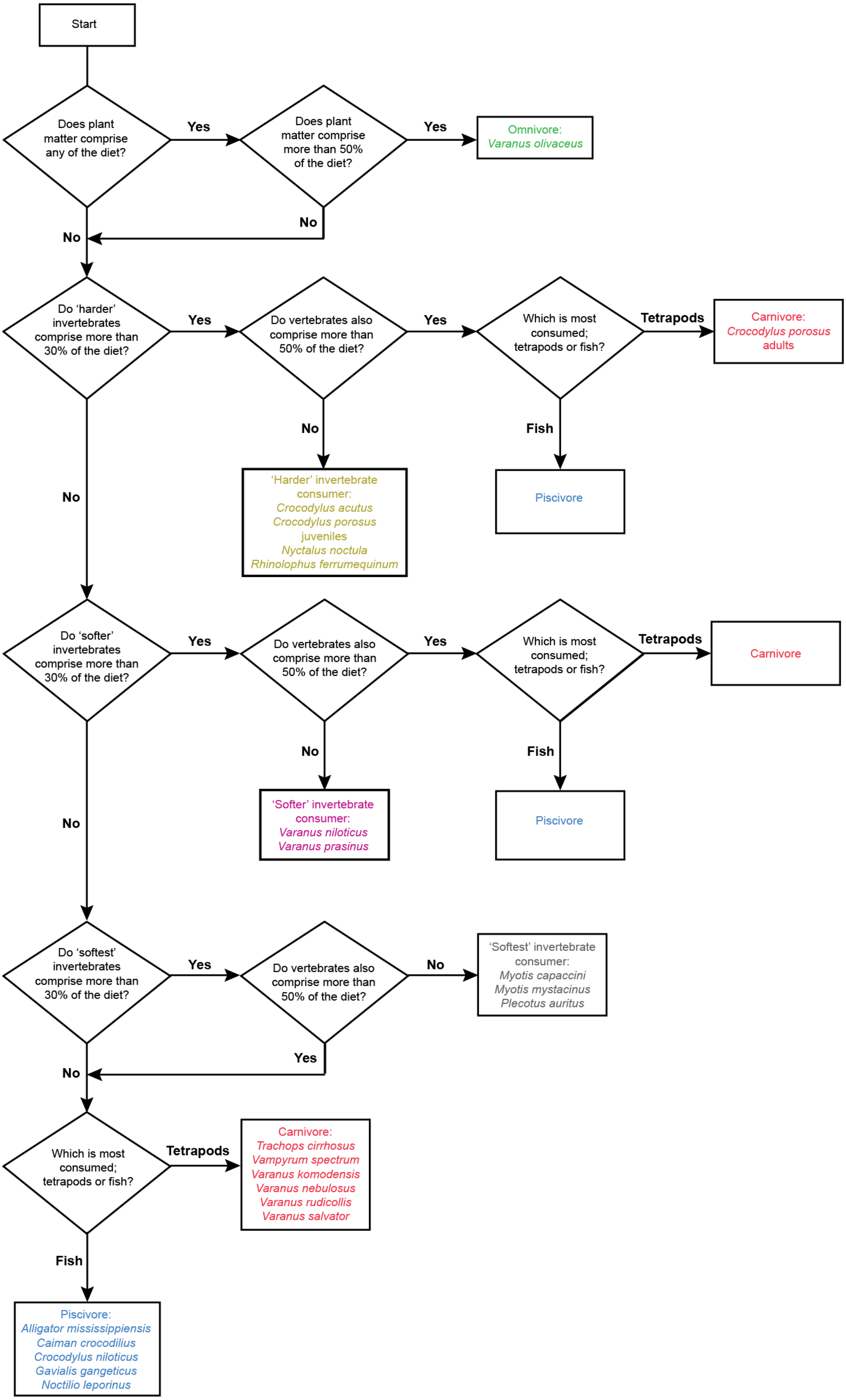
Start
Does plant matter comprise any of the diet?
Yes
Does plant matter comprise more than 50% of the diet?
Yes
Omnivore: Varanus olivaceus
No
No
Do 'harder' invertebrates comprise more than 30% of the diet?
Yes
Do vertebrates also comprise more than 50% of the diet?
Yes
Which is most consumed; tetrapods or fish?
Tetrapods
Carnivore: Crocodylus porosus adults
No
Fish
'Harder' invertebrate consumer: Crocodylus acutus Crocodylus porosus juveniles Nyctalus noctula Rhinolophus ferrumequinum
Piscivore
No
Do 'softer' invertebrates comprise more than 30% of the diet?
Yes
Do vertebrates also comprise more than 50% of the diet?
Yes
Which is most consumed; tetrapods or fish?
Tetrapods
Carnivore
No
Fish
'Softer' invertebrate consumer: Varanus niloticus Varanus prasinus
Piscivore
No
Do 'softest' invertebrates comprise more than 30% of the diet?
Yes
Do vertebrates also comprise more than 50% of the diet?
No
'Softest' invertebrate consumer: Myotis capaccini Myotis mystacinus Plecotus auritus
Yes
No
Which is most consumed; tetrapods or fish?
Tetrapods
Carnivore: Trachops cirrhosus Vampyrum spectrum Varanus komodensis Varanus nebulosus Varanus rudicollis Varanus salvator
Fish
Piscivore: Alligator mississippiensis Caiman crocodilius Crocodylus niloticus Gavialis gangeticus Noctilio leporinus

[Previous page] **Supplementary Fig. 1 | Schematic overview classifying extant reptiles and bats into respective dietary guilds.** Figure adapted from Bestwick et al.[2] under a Creative Commons Attribution 4.0 International License (https://creativecommons.org/licenses/by/4.0/) to include the guild classifications of the sampled bat species. Dietary information derived from stomach and/or faecal content studies which provide dietary constituents as volumetric or frequency proportions of total diet. See Methods for classification details and Supplementary Data 2[1] for proportional breakdowns of reptile and bat diets and for the studies from which they are derived.

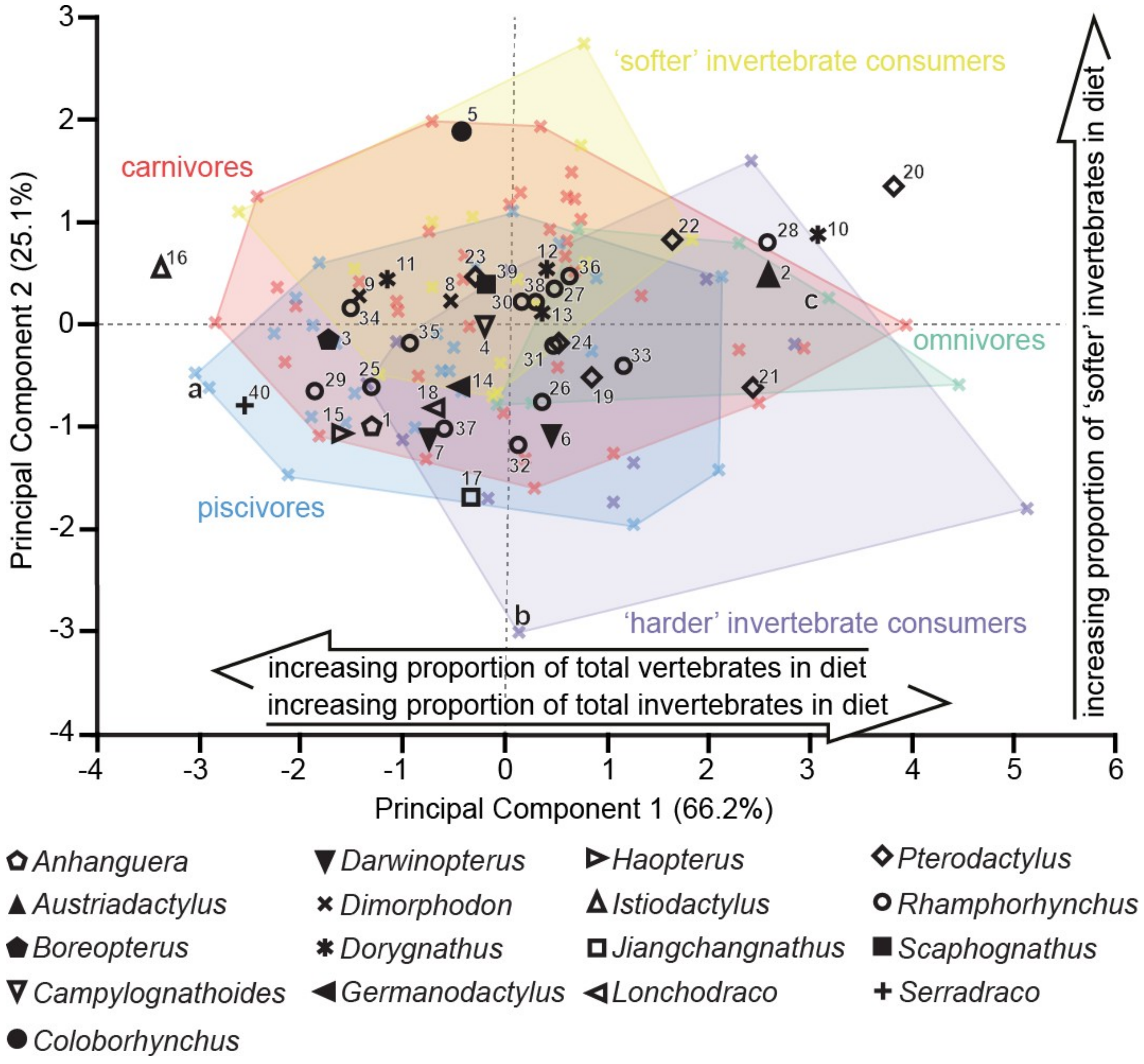

Supplementary Fig. 2 | Texture-dietary space of reptiles and pterosaurs with pterosaur PCA plot numbers. Pterosaur specimen information corresponding to plot number can be found in Supplementary Data 1[1]. Texture-dietary space adapted from fig. 2 of Bestwick et al.[2] (https://www.nature.com/articles/s41598-019-48154-9/figures/2) by Jordan Bestwick under a Creative Commons Attribution 4.0 International License (https://creativecommons.org/licenses/by/4.0/) to include the pterosaur data.

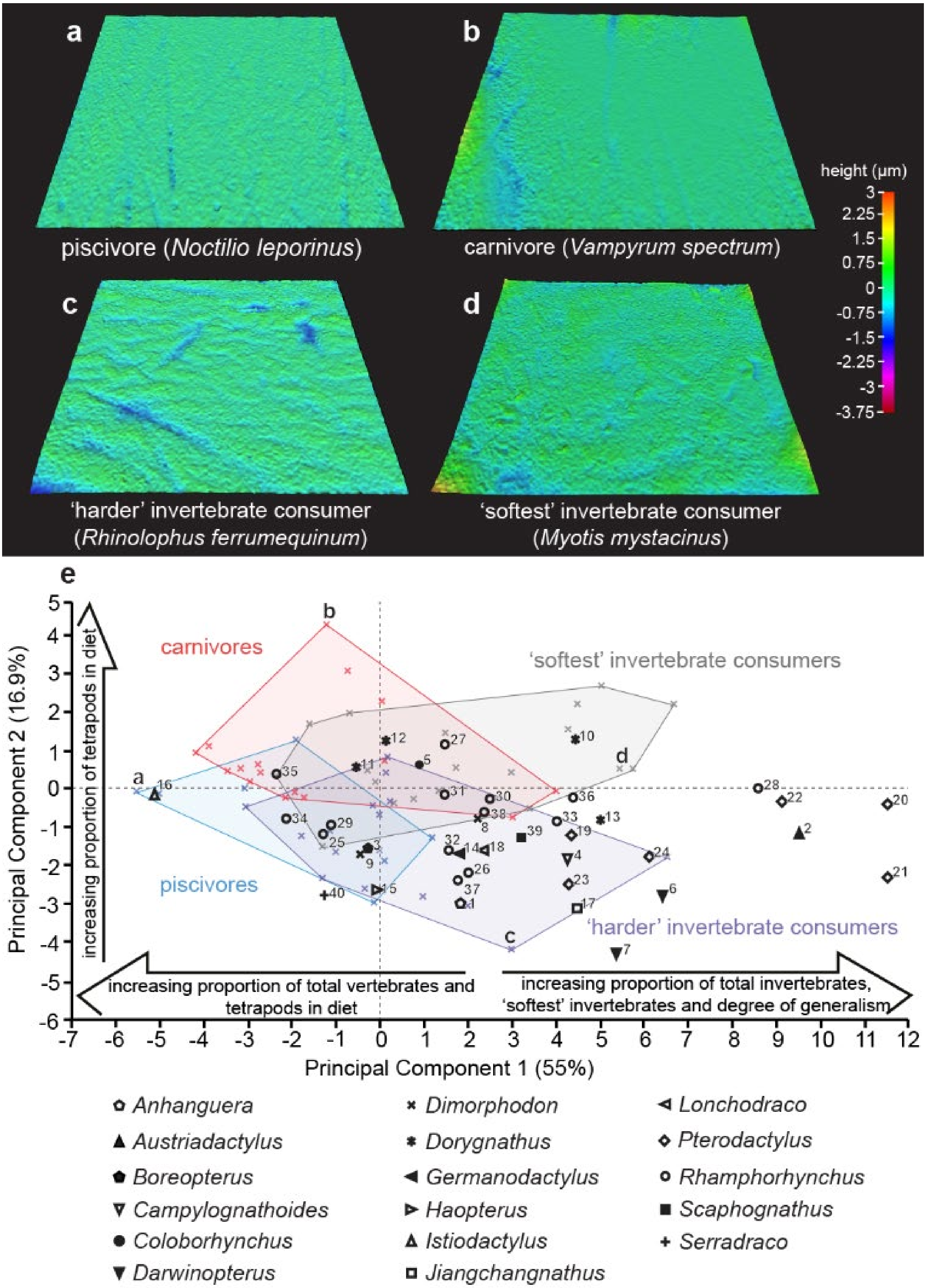
a
piscivore (Noctilio leporinus)
b
carnivore (Vampyrum spectrum)
height (µm)
3
2.25
1.5
0.75
0
-0.75
-1.5
-2.25
-3
-3.75
c
'harder' invertebrate consumer (Rhinolophus ferrumequinum)
d
'softest' invertebrate consumer (Myotis mystacinus)
e
carnivores
'softest' invertebrate consumers
piscivores
'harder' invertebrate consumers
Principal Component 2 (16.9%)
increasing proportion of tetrapods in diet
increasing proportion of total vertebrates and tetrapods in diet
increasing proportion of total invertebrates, 'softest' invertebrates and degree of generalism
Principal Component 1 (55%)
Anhanguera
Austriadactylus
Boreopterus
Campylognathoides
Coloborhynchus
Darwinopterus
Dimorphodon
Dorygnathus
Germanodactylus
Haopterus
Istiodactylus
Jiangchangnathus
Lonchodraco
Pterodactylus
Rhamphorhynchus
Scaphognathus
Serradraco

[Previous page] **Supplementary Fig. 3 | Quantitative textural analysis of microwear in extant bats and pterosaurs**. (**a**–**d**), Example scale-limited surfaces of bat dietary guilds: (**a**) piscivore (*Noctilio leporinus;* greater bulldog bat), (**b**) carnivore (*Vampyrum spectrum*; spectral bat), (**c**) ‘softest’ invertebrate consumer (*Myotis mystacinus*; whiskered bat) and (**d**) ‘harder’ invertebrate consumer (*Rhinolophus ferrumequinum*; greater horseshoe bat). Measured areas 146 X 110 µm in size. Topographic scale in micrometres. (**e**) Texture-dietary space of International Organisation for Standardisation (ISO) texture parameters from bats and pterosaurs. Texture-dietary space based on extant bat data (*n* = 59) with pterosaurs projected onto the first two axes as unknown datum points. Arrows show significant correlations of dietary characteristics along PC axes 1 and 2. Specimens with associated letters represent surfaces (**a**–**d**). Pterosaur PCA plot numbers same as Supplementary Fig. 2. Pterosaur specimen information corresponding to PCA plot number can found in the Supplementary Data 1[1].

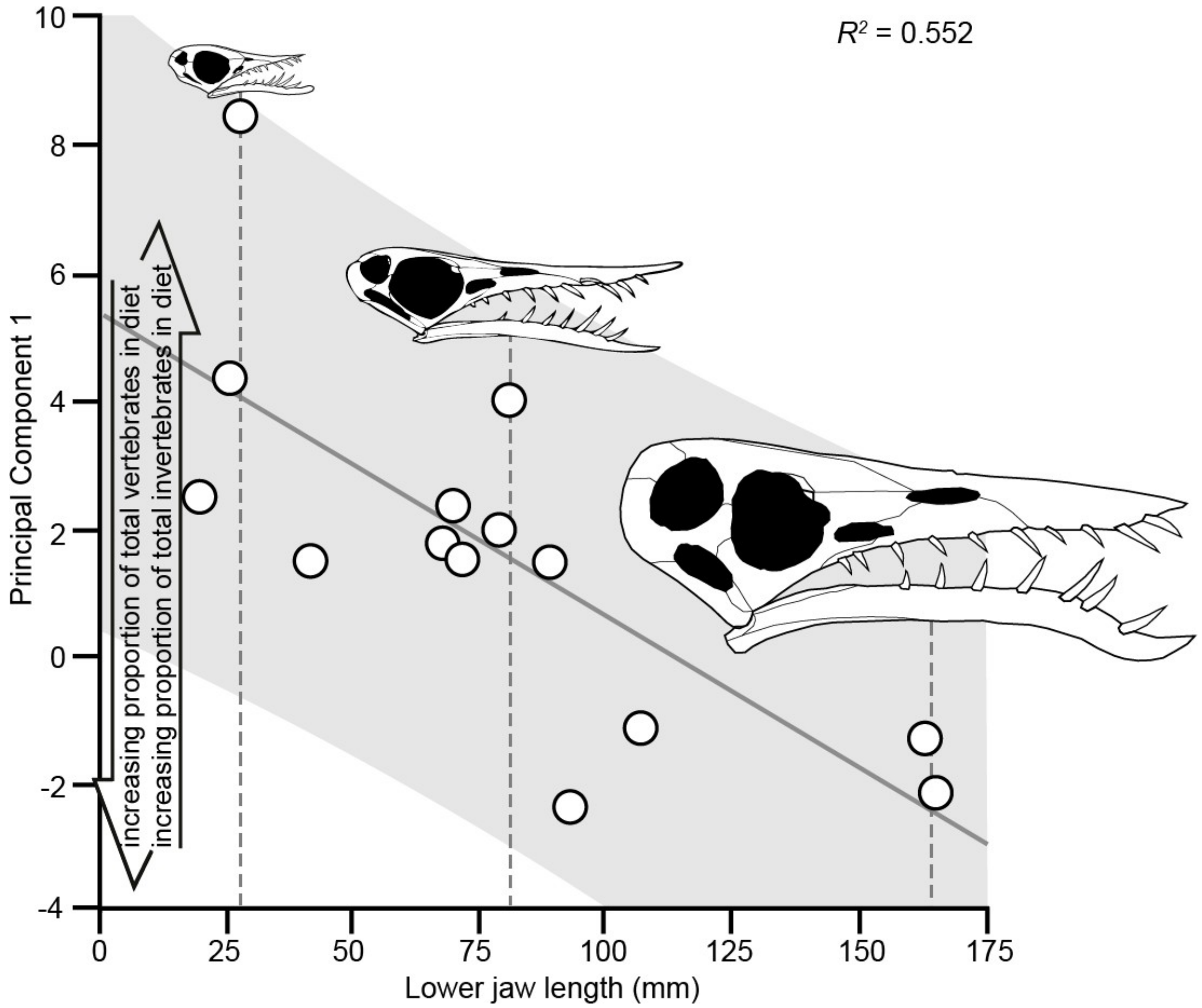

Supplementary Fig. 4 | *Rhamphorhynchus* ontogenetic shifts in the bat texture-dietary space. Regression of lower jaw length (*n* = 14), as a proxy for specimen size, plotted against PC 1 values from the extant bat texture-dietary space. The solid grey line denotes the line of best fit and grey shading denotes the 95% confidence intervals. The largest to smallest skull diagrams represent scaled examples of an adult, juvenile and hatchling *Rhamphorhynchus*, with the dashed grey lines denoting their relative size. These diagrams illustrate the size disparity between sampled life-history stages and are not specimen reconstructions of the datum points they are associated with. See Methods for the skull diagram sources.

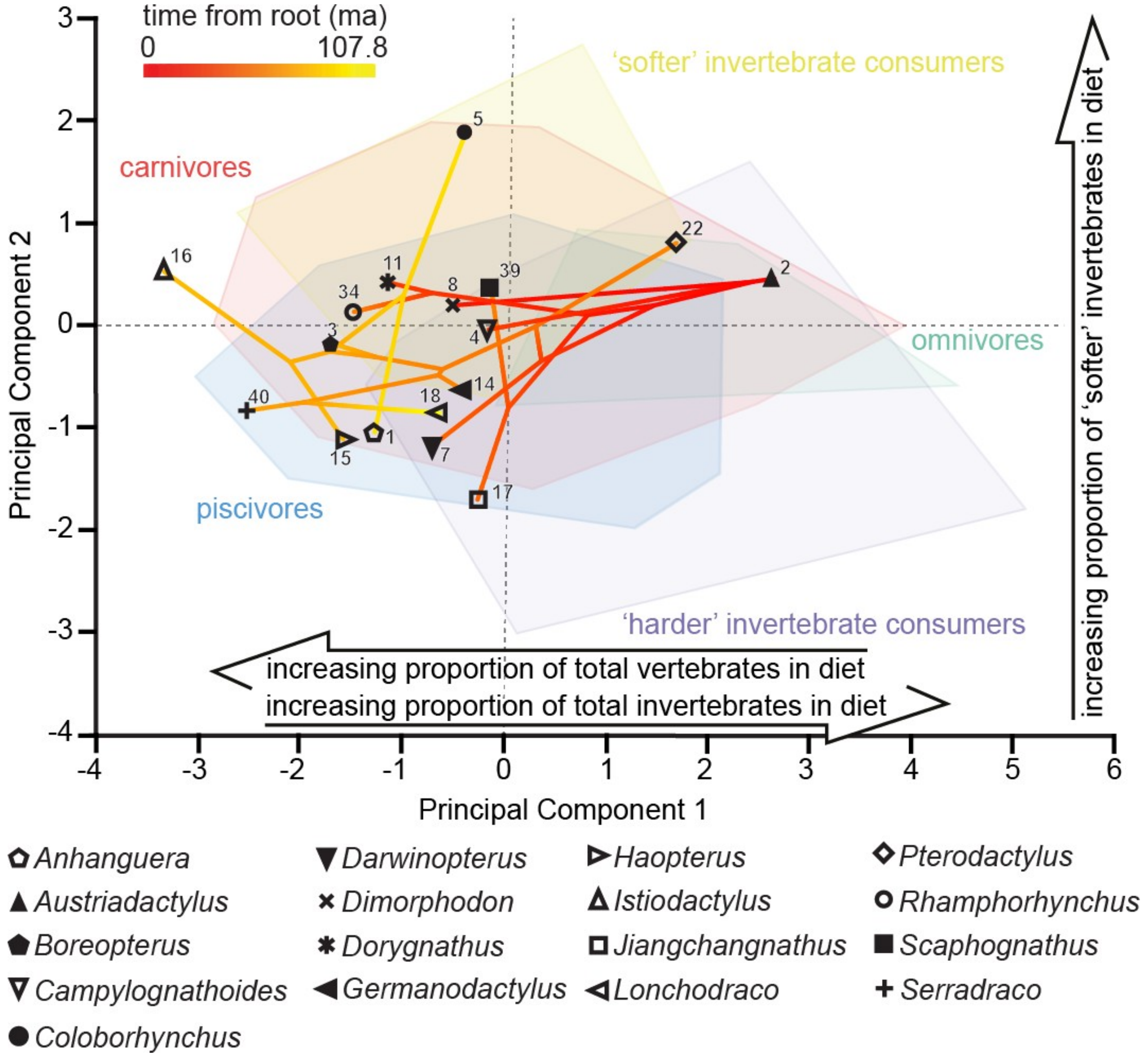

Supplementary Fig. 5 | "Phylo-texture-dietary space" of pterosaur microwear data using the pruned tree from Lü et al.[15] with pterosaur PCA plot numbers. Pterosaur specimen information corresponding to PCA plot number can found in Supplementary Data 1[1]. Phylo-texture-dietary space adapted from fig. 2 of Bestwick et al.[2] (https://www.nature.com/articles/s41598-019-48154-9/figures/2) by Jordan Bestwick under a Creative Commons Attribution 4.0 International License (https://creativecommons.org/licenses/by/4.0/) to remove the extant reptile datum points and to include the pterosaur data and phylogeny.

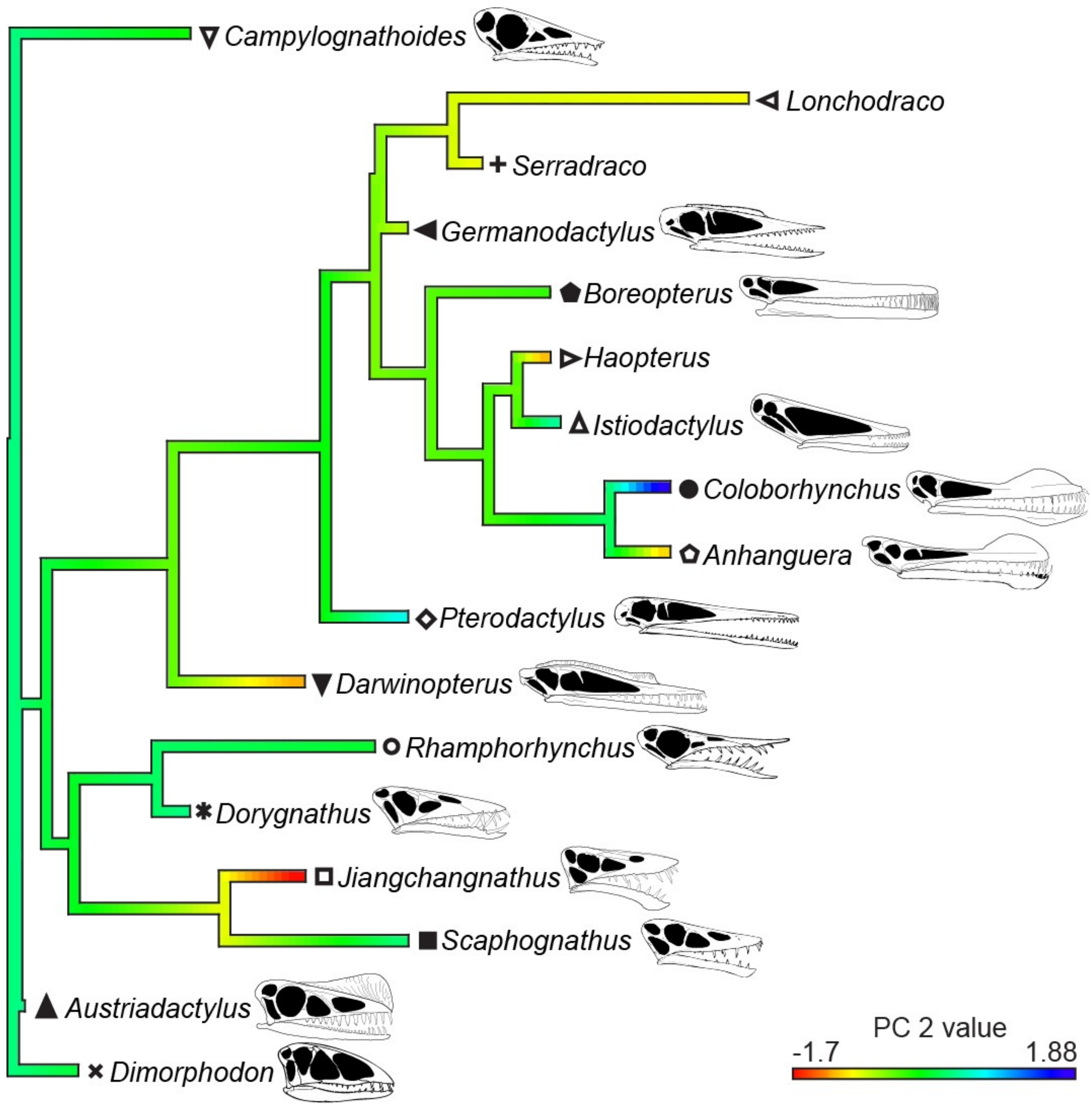

Supplementary Fig. 6 | Ancestral character-state reconstruction of pterosaur diets from mapping PC 2 values onto a time-calibrated pruned tree from Lü et al.[15]. Pterosaur PC 2 values from the extant reptile texture-dietary space. Only the largest specimen for each pterosaur species was included. Symbols denoting pterosaurs same as in Fig. 4. Skull diagrams of well-preserved pterosaurs not to scale (see Methods for sources).

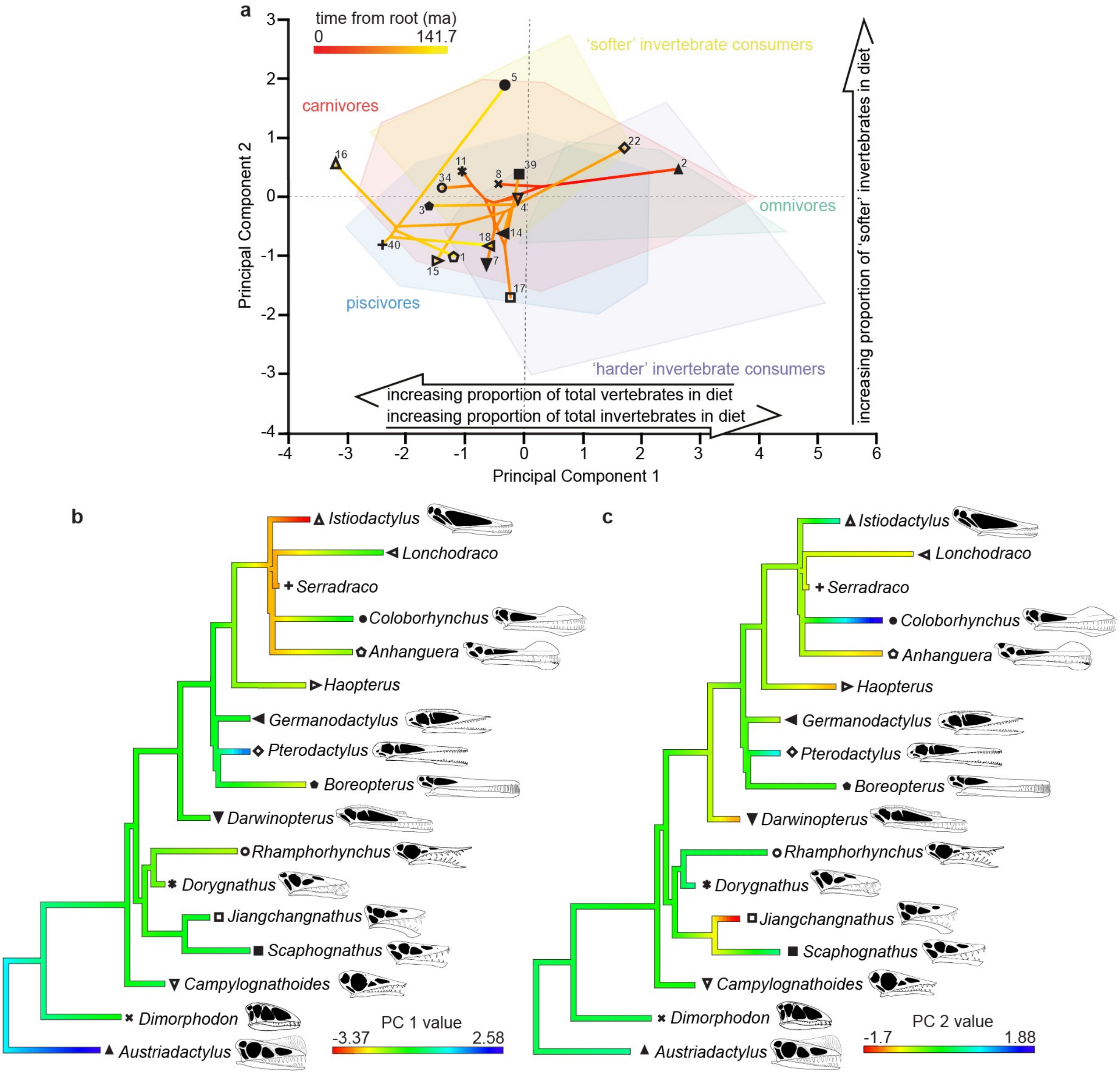

Supplementary Fig. 7 | Pterosaur dietary evolution using a time-calibrated pruned tree from Andres & Myers[13]. (**a**) **"**Phylo-texture-dietary space" of pterosaur microwear data, formed by projecting the tree onto the first two PC axes of the extant reptile texture-dietary space. (**b**) Ancestral character-state reconstruction of pterosaur dietary evolution from mapping pterosaur PC 1 values onto the tree. (**c**) Ancestral character-state reconstruction from mapping pterosaur PC 2 values onto the tree. Only the largest specimen of each pterosaur species was included. Pterosaur symbols and PCA plot numbers same as Fig. 4 and Supplementary Fig. 5. Skull diagrams of well- preserved pterosaurs not to scale (see Methods for sources). Phylo-

texture-dietary space adapted from fig. 2 of Bestwick et al.[2] (https://www.nature.com/articles/s41598-019-48154-9/figures/2) by Jordan Bestwick under a Creative Commons Attribution 4.0 International License (https://creativecommons.org/licenses/by/4.0/) to remove the extant reptile datum points and to include the pterosaur data and phylogeny.

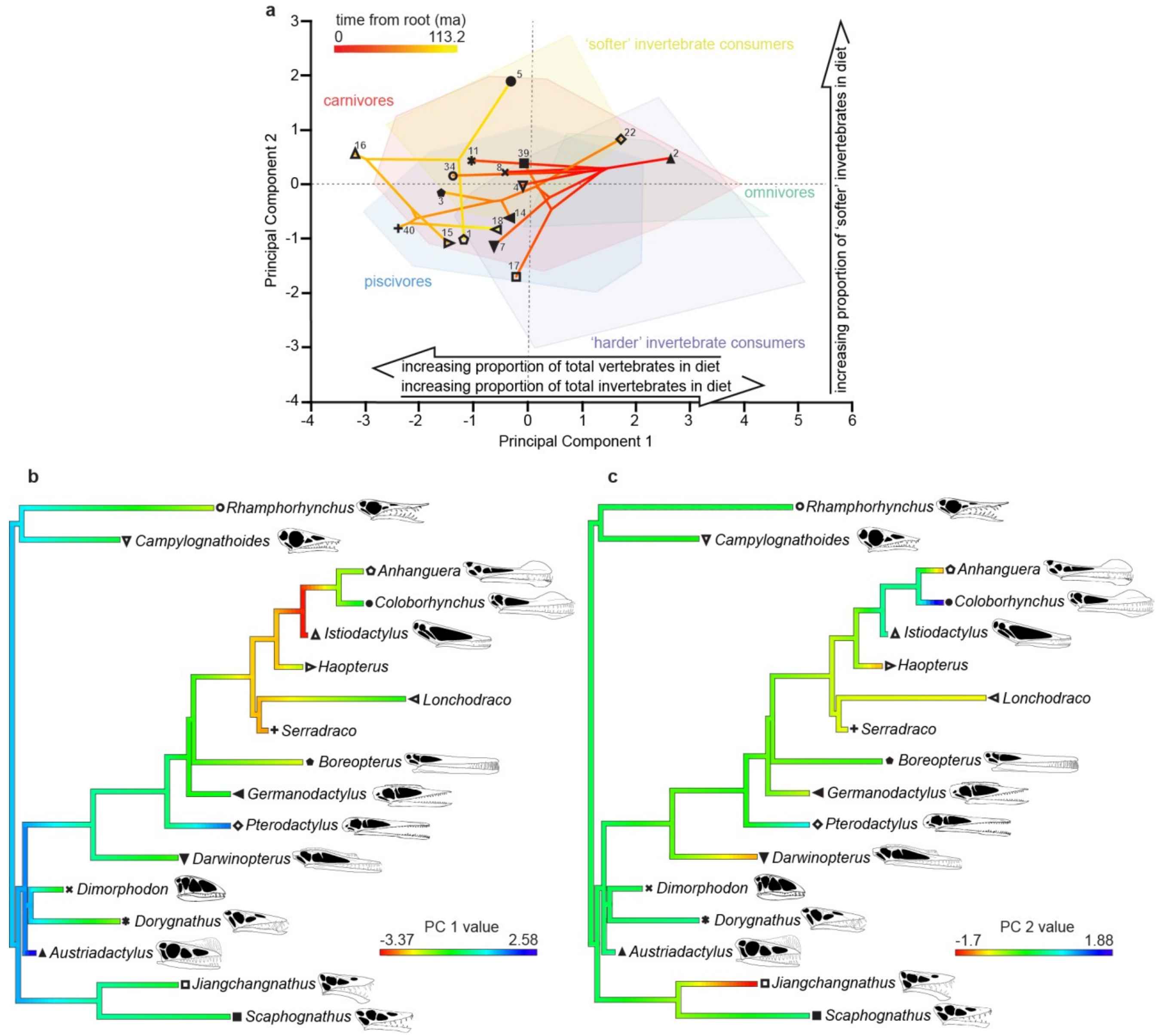

Supplementary Fig. 8 | Pterosaur dietary evolution using a time-calibrated pruned tree from Wang et al.[14]. (**a**) **"**Phylo-texture-dietary space" of pterosaur microwear data, formed by projecting the tree onto the first two PC axes of the extant reptile texture-dietary space. (**b**) Ancestral character-state reconstruction of pterosaur dietary evolution from mapping pterosaur PC 1 values onto the tree. (**c**) Ancestral character-state reconstruction from mapping pterosaur PC 2 values onto the tree. Only the largest specimen of each pterosaur species was included. Pterosaur symbols and PCA plot numbers same as Fig. 4 and Supplementary Fig. 5. Skull diagrams of well-preserved pterosaurs not to scale (see Methods for sources). Phylo-texture-dietary space adapted from fig. 2 of Bestwick et al.[2] (https://www.nature.com/articles/s41598-

019-48154-9/figures/2) by Jordan Bestwick under a Creative Commons Attribution 4.0 International License (https://creativecommons.org/licenses/by/4.0/) to remove the extant reptile datum points and to include the pterosaur data and phylogeny.

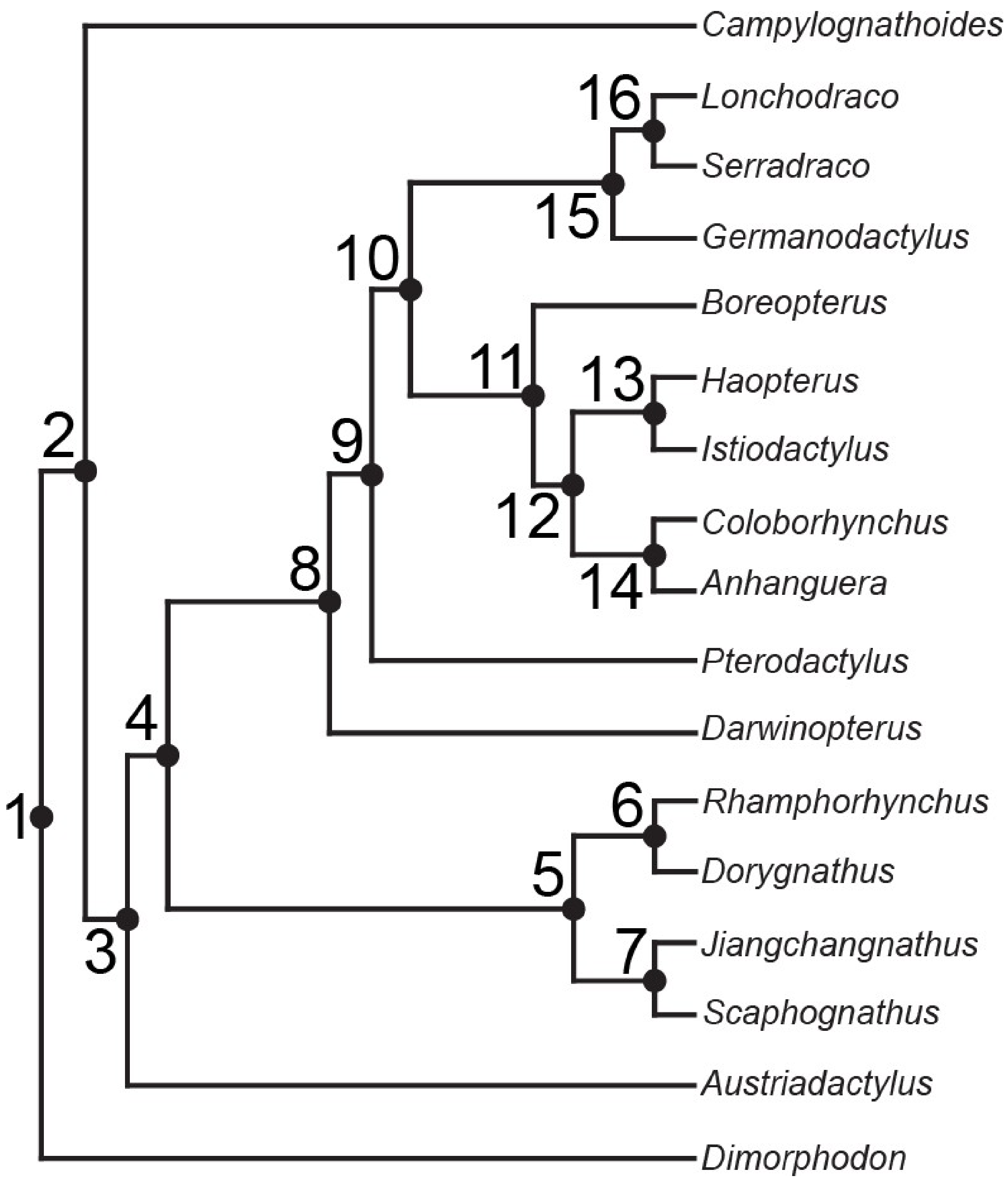

Supplementary Fig. 9 | Non-time-calibrated pruned tree from Lü et al.[15] showcasing the node positions in the ancestral pterosaur dietary state reconstructions. See Supplementary Data 6[1] for the estimated PC 1 and 2 values, as well as the variance and 95% confidence intervals, for each labelled node. Nodes: 1, Pterosauria; 2, Node 3 + *Campylognathoides*; 3, Node 4 + *Austriadactylus*; 4, Monofenestrata + Rhamphorhynchidae; 5, Rhamphorhynchidae; 6, Rhamphorhynchinae; 7, Scaphognathinae; 8, Monofenestrata; 9; Pterodactyloidea; 10, Node 15 + Ornithocheiroidea; 11, Ornithocheiroidea; 12, Istiodactylidae + Ornithocheiridae; 13, Istiodactylidae; 14, Ornithocheiridae; 15, Lonchodectidae + *Germanodactylus*; 16, Lonchodectidae.

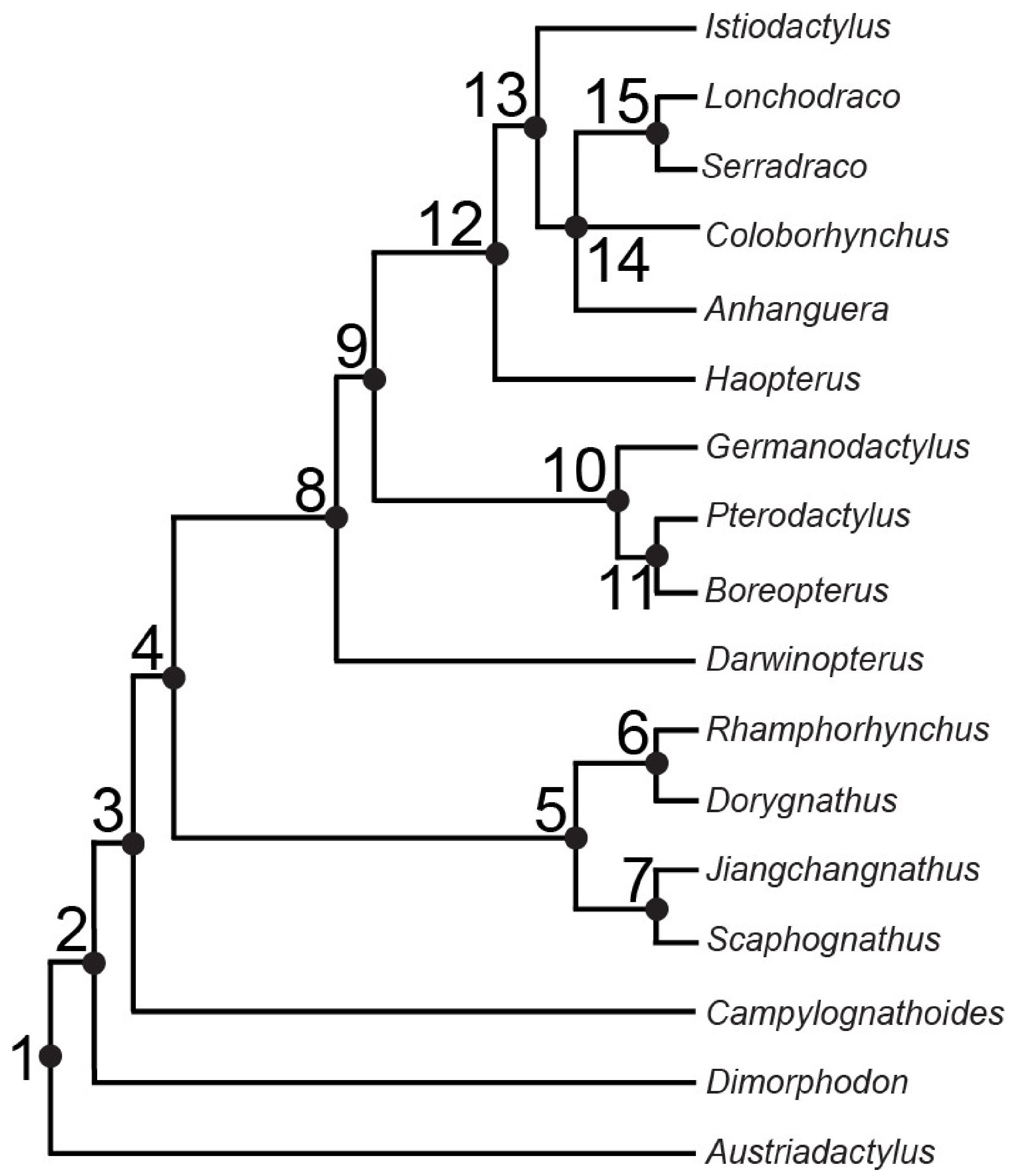

Supplementary Fig. 10 | Non-time-calibrated pruned tree from Andres & Myers[13] showcasing the node positions in the ancestral pterosaur dietary state reconstructions. See Supplementary Data 6[1] for the estimated PC 1 and 2 values, as well as the variance and 95% confidence intervals, for each labelled node. Nodes: 1, Pterosauria; 2, Macronychoptera; 3, Novialoidea; 4, Breviquartossa; 5, Rhamphorhynchidae; 6, Rhamphorhynchinae; 7, *Jiangchangnathus* + *Scaphognathus*; 8, Monofenestrata; 9, Pterodactyloidea; 10, Archaeopterodactyloidea; 11, Euctenochasmatia; 12, Eupterodactyloidea; 13, Ornithocheiroidea; 14, Anhangueria; 15, Lonchodectidae.

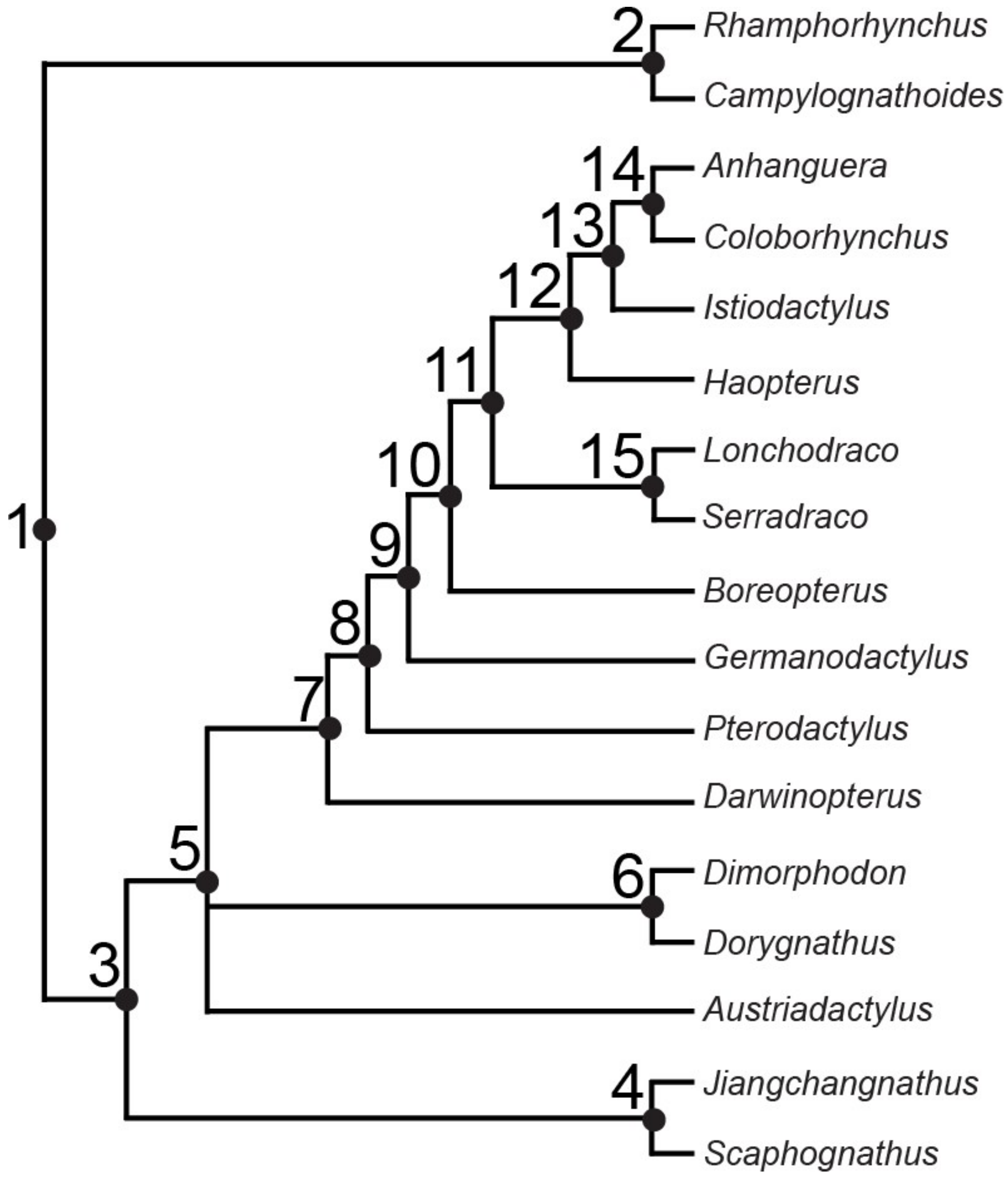

Supplementary Fig. 11 | Non-time-calibrated pruned tree from Wang et al.[14] showcasing the node positions in the ancestral pterosaur dietary state reconstructions. See Supplementary Data 6[1] for the estimated PC 1 and 2 values, as well as the variance and 95% confidence intervals, for each labelled node. Nodes: 1; Pterosauria; 2, *Rhamphorhynchus* + *Campylognathoides*; 3, Node 4 + Node 5; 4, *Jiangchangnathus* + *Scaphognathus*; 5, Monofenestrata + (*Dimorphodon* + *Dorygnathus*) + *Austriadactylus*; 6, *Dimorphodon* + *Dorygnathus*; 7, Monofenestrata; 8, Pterodactyloidea; 9, Node 10 + *Germanodactylus*; 10, Pteranodontoidea; 11, Lanceodontia; 12, Node 13 + *Haopterus*; 13, Node 14 + *Istiodactylus*; 14, Anhangueridae; 15, *Lonchodraco* + *Serradraco*.

Supplementary Tables

Supplementary Table 1 | ISO texture parameter average value rankings between bat dietary guilds. 1 = most positive parameter values, 5 = least positive values. Parameters in bold are used in the principal component analysis (Supplementary Fig. 2). Average rank of all parameters per guild to 4 d.p. Abbreviations: C, Carnivores; HI; 'Harder' Invertebrate consumers, P: Piscivores, StI; 'Softest' Invertebrate consumers. See Supplementary Data 4[1] for ISO parameter definitions.

Parameter	C	HI	P	StI
**Sq**	**4**	**2**	**3**	**1**
Sku	3	2	4	1
**Sp**	**3**	**2**	**4**	**1**
**Sv**	**4**	**2**	**3**	**1**
**Sz**	**3**	**2**	**4**	**1**
**Sds**	**1**	**4**	**3**	**2**
**Str**	**2**	**3**	**4**	**1**
Sdq	2	3	4	1
**Ssc**	**2**	**4**	**3**	**1**
Sdr	2	3	4	1
**Vmp**	**3**	**2**	**4**	**1**
**Vmc**	**4**	**1**	**3**	**2**
**Vvc**	**4**	**2**	**3**	**1**
Vvv	4	1	3	2
**Spk**	**3**	**2**	**4**	**1**
**Sk**	**4**	**1**	**3**	**2**
Svk	4	1	3	2
**Smr1**	**2**	**4**	**3**	**1**
Smr2	2	3	4	1
**S5z**	**3**	**2**	**4**	**1**
**Sa**	**4**	**2**	**3**	**1**

Supplementary Table 2 | Matched-pairs t-test results (4 d.p) of the means of ISO texture parameters between bat dietary guilds. Data log transformed and scale limited using 5th order polynomial and robust Gaussian filter. Pairwise tests exhibiting significant differences after application of the Benjamini-Hochberg procedure shown in bold. All tests were two-tailed. Abbreviations: C, Carnivores; HI; 'Harder' invertebrate consumers, P; Piscivores, StI; 'Softest' invertebrate consumers.

Pairwise test	*t*-ratio	*P*-value	d.f
C-HI	2.0574	0.0529	20
C-P	-2.1524	0.438	20
**C-StI**	**6.2578**	**<0.0001**	**20**
**HI-P**	**-4.7816**	**0.0001**	**20**
**HI-StI**	**4.7205**	**0.0001**	**20**
**P-StI**	**5.8922**	**<0.0001**	**20**

Supplementary References

1. Bestwick, J., Unwin, D. M., Butler, R. J. & Purnell, M. A. Dietary diversity and evolution of the earliest flying vertebrates revealed by dental microwear texture analysis Datasets. *Zenodo* https://doi.org/10.5281/zenodo.4018876
2. Bestwick, J., Unwin, D. M. & Purnell, M. A. Dietary differences in archosaur and lepidosaur reptiles revealed by dental microwear textural analysis. *Sci. Rep.* **9**, 11691 (2019).
3. Winkler, D. E., Schulz-Komas, E., Kaiser, T. M. & Tütken, T. Dental microwear texture reflects dietary tendencies in extant Lepidosauria despite their limited use of oral food processing. *Proc. R. Soc. B.* **286**, 20190544 (2019).
4. Witton, M. P. New insights into the skull of *Istiodactylus latidens* (Ornithocheiroidea, Pterodactyloidea). *PLoS ONE* **7**, e33170 (2012).
5. Bestwick, J., Unwin, D. M., Butler, R. J., Henderson, D. M. & Purnell, M. A. Pterosaur dietary hypotheses: a review of ideas and approaches. *Biol. Rev.* **93**, 2021–2048 (2018).
6. Sah, S. A. M. & Stuebing, R. B. Diet, growth and movements of juvenile crocodiles *Crocodylus porsus* Schneider in the Klias river, Sabah, Malaysia. *J. Trop. Ecol.* **12**, 651–662 (1996).
7. Auffenberg, W. *Gray's Monitor Lizard*. (Univ. Press. Florida, 1988).
8. Flanders, F. & Jones, G. Roost use, ranging behavior, and diet of greater horseshoe bats (*Rhinolophus ferrumequinum*) using a transitional roost. *J. Mammal.* **90**, 888–896 (2009).
9. Bennett, S. C. A statistical study of *Rhamphorhynchus* from the Solnhofen limestone of Germany: year-classes of a single large species. *J. Paleontol.* **69**, 569–580 (1995).
10. Prondvai, E., Stein, K., Ősi, A. & Sander, M. P. Life history of *Rhamphorhynchus* inferred from bone histology and the diversity of pterosaurian growth strategies. *PLoS One* **7**, e31392 (2012).
11. Bennett, S. C. New smallest specimen of the pterosaur *Pteranodon* and ontogenetic niches in pterosaurs. *J. Vert. Paleontol.* **92**, 254–271 (2017).
12. Unwin, D. M. *The Pterosaurs from Deep Time*. (Pi Press, 2006).
13. Andres, B. & Myers, T. S. Lone star pterosaurs. *Earth Env. Sci. T. R. So.* **103**, 383–398 (2013).
14. Wang, X. et al. New evidence from China for the nature of the pterosaur evolutionary transition. *Sci. Rep.* **7**, 42763 (2017).

15. Lü, J., Kundrát, M. & Shen, C. New material of the pterosaur *Gladocephaloideus* Lü et al., 2012 from the Early Cretaceous of Liaoning Province, China, with comments on its systematic position. *PLoS One* **11**, e0154888 (2016).

16. Ősi, A. Feeding-related characters in basal pterosaurs: implications for jaw mechanism, dental function and diet. *Lethaia* **44**, 136–152 (2011).

17. Zhou, C.-F. et al. Earliest filter-feeding pterosaur from the Jurassic of China and ecological evolution of Pterodactyloidea. *Royal Soc. Open Sci.* **4**, 160672 (2017).

18. Dalla Vecchia, F. M. in *Anatomy, phylogeny and palaeobiology of early archosaurs and their kin* (eds Nesbitt, S. J., Desojo, J. B. & Irmis, R. B.) **379**, 119–155 (Geol. Soc. Lond. Spec. Pub., 2013).

19. Andres, B. Death and taxa: the shape of pterosaur evolution. In *The 5th International Symposium on Pterosaurs.* (Univ. Portsmouth, UK, 2015).

20. Benson, R. B. J. & Choiniere, J. N. Rates of dinosaur limb evolution provide evidence for exceptional radiation in Mesozoic birds. *Proc. R. Soc. B.* **280,** 20131780 (2013).

21. Mitchell, J. S. & Makovicky, P. J. Low ecological disparity in Early Cretaceous birds. *Proc. R. Soc. B.* **281,** 20140608 (2014).
