# Supplementary material for: Dietary diversity and evolution of the earliest flying vertebrates revealed by dental microwear texture analysis
Source: Nat Commun. 2020 Oct 28;11:5293. doi: 10.1038/s41467-020-19022-2 (PMC7595196; doi:10.1038/s41467-020-19022-2)
Supplement: Supplementary file 3 — Reporting Summary [file 41467_2020_19022_MOESM3_ESM.pdf]

nature research

Corresponding author(s): Jordan Bestwick, Mark Purnell

Last updated by author(s): Sep 9, 2020

Reporting Summary

Nature Research wishes to improve the reproducibility of the work that we publish. This form provides structure for consistency and transparency in reporting. For further information on Nature Research policies, see our Editorial Policies and the Editorial Policy Checklist.

Statistics

For all statistical analyses, confirm that the following items are present in the figure legend, table legend, main text, or Methods section.

n/a	Confirmed	
☐	☒	The exact sample size (*n*) for each experimental group/condition, given as a discrete number and unit of measurement
☐	☒	A statement on whether measurements were taken from distinct samples or whether the same sample was measured repeatedly
☐	☒	The statistical test(s) used AND whether they are one- or two-sided<br>*Only common tests should be described solely by name; describe more complex techniques in the Methods section.*
☐	☒	A description of all covariates tested
☐	☒	A description of any assumptions or corrections, such as tests of normality and adjustment for multiple comparisons
☐	☒	A full description of the statistical parameters including central tendency (e.g. means) or other basic estimates (e.g. regression coefficient) AND variation (e.g. standard deviation) or associated estimates of uncertainty (e.g. confidence intervals)
☐	☒	For null hypothesis testing, the test statistic (e.g. *F*, *t*, *r*) with confidence intervals, effect sizes, degrees of freedom and *P* value noted<br>*Give P values as exact values whenever suitable.*
☒	☐	For Bayesian analysis, information on the choice of priors and Markov chain Monte Carlo settings
☒	☐	For hierarchical and complex designs, identification of the appropriate level for tests and full reporting of outcomes
☐	☒	Estimates of effect sizes (e.g. Cohen's *d*, Pearson's *r*), indicating how they were calculated

Our web collection on statistics for biologists contains articles on many of the points above.

Software and code

Policy information about availability of computer code

Data collection	Two pieces of commercially available software were used to produce microwear texture data and the scale-limited texture models: (i) Alicona GMbH, Graz, Austria, software version 5.1 (this was already installed on the Alicona Infinite Focus microscope G4b at the University of Leicester, UK), (ii) Surfstand 5.0.0, Centre for Precision Technologies, University of Huddersfield, West Yorkshire, UK)
Data analysis	(i) the commercially available software JMP Pro 12 (SAS Institute, Cary, NC, USA) was used to analyse the microwear texture data. We utilised an institutional license for the software package that was bought by the University of Leicester, UK. (ii) Microsoft Corporation, 2016. Microsoft Excel, Available at: https://office.microsoft.com/excel (iii) the free to use Benjamini-Hochberg correction Microsoft Excel sheet www.biostathandbook.com/multiplecomparisons.html was also used to analyse the microwear texture data. (iv) the free to use software R version 4.0.0 was used in the dietary evolution analyses. The custom code that was written for the dietary evolution analyses is submitted as the R file "Supplementary Code 1". (v) the free to use R package ape, version 5.4-1 (vi) the free to use R package phytools, version 0.6-60 (vii) the free to use R package strap, version 1.4

For manuscripts utilizing custom algorithms or software that are central to the research but not yet described in published literature, software must be made available to editors and reviewers. We strongly encourage code deposition in a community repository (e.g. GitHub). See the Nature Research guidelines for submitting code & software for further information.

Data

Policy information about availability of data

All manuscripts must include a data availability statement. This statement should provide the following information, where applicable:

- Accession codes, unique identifiers, or web links for publicly available datasets
- A list of figures that have associated raw data
- A description of any restrictions on data availability

Data availability statement: All the data analysed in this study are publically available in Zenodo (https://doi.org/10.5281/zenodo.4018876) and within the Supplementary Information files.

Field-specific reporting

Please select the one below that is the best fit for your research. If you are not sure, read the appropriate sections before making your selection.

☐ Life sciences ☐ Behavioural & social sciences ☒ Ecological, evolutionary & environmental sciences

For a reference copy of the document with all sections, see nature.com/documents/nr-reporting-summary-flat.pdf

Ecological, evolutionary & environmental sciences study design

All studies must disclose on these points even when the disclosure is negative.

Study description	We reconstructed the diets of pterosaurs – extinct flying reptiles that lived between 210 and 66 million years ago – by quantitative analysis of their microscopic scale tooth surface textures that formed during food consumption, known as dental microwear texture analysis. To provide modern frameworks for dietary reconstructions we also sampled the tooth microwear textures of modern bats with known diets, based on previously published stomach or faecal content studies. We also utilised the microwear dataset of modern crocodilians and monitor lizards from Bestwick et al. (2019) Sci. Rep. 9, 11091 to serve as an independent framework to the bats. Each bat and reptile species was assigned to a dietary guild based on the relative proportions of consumed foods and the material properties of food items (e.g. carnivore, harder invertebrate consumer etc.). Tooth texture data were generated for each sampled tooth from 22 International Organisation for Standardisation 25178-2 areal texture parameters (e.g. average peak height, average trough depth). One-way ANOVAs with pairwise testing (Tukey HSD) was applied to each texture parameter to test for microwear differences between guilds. Principal Component Analysis was performed with paramters that significantly differed between guilds. We then performed Spearman's rank to test for correlations between PC axes 1 and 2 with dietary and textural differences. Matched pairs t-tests were performed on the average values of each parameter between guilds. Pterosaur microwear data were then independently projected into the PCA of the reptiles and of the bats as unknown datum points to see where they would plot in the PCA, from which the likely diets were reconstructed. We used pterosaur placements within the reptile PCA to reconstruct pterosaur dietary evolution. This was done by projected time-calibrated phylogenetic trees from Lu et al. (2016) PLoS ONE, 11, e0154888, doi:10.1371/journal.pone.0154888, Andres & Myers (2013) Earth Env. Sci. T. R. Soc. Edinburgh. 103, 383–398 doi:10.1017/s1755691013000303 and, Wang et al. (2017) Sci. Rep. 7, 42763 into the modern reptile PCA. The pterosaur PCA data were then projected into each of these pruned phylogenies. All statistical procedures are thoroughly described in the methods section of the manuscript.
Research sample	40 specimens from 17 genera comprised the pterosaur dataset. Due to museum availability and specimen preservation quality (only teeth with the original enamel preserved were sampled), some genera are only represented by one or two specimens which limits the strength of the conclusions that can be drawn from the dietary reconstructions of these genera. This caveat is mentioned in the main and supporting texts. Other pterosaur genera are represented by at least several specimens which enables more constrained conclusions to be drawn from these dietary reconstructions. The bat dataset comprises 59 specimens of dry skeletons from eight species. These eight species were chosen due to having well-constrained diets from previous faecal content studies. Sample sizes of guilds were largely determined by museum availability and specimen preservation quality. Only adults were sampled no preference was given for sex. This study also utilised the modern reptile microwear dataset of Bestwick et al. (2019) Sci Rep. 9, 11691, doi: 10.1038/s41598-019-48154-9 which comprises 95 specimens from six species of crocodilian and seven species of monitor lizard. For our dietary evolution reconstructions we utilise the phylogenies of Lu et al. (2016) PLoS ONE, 11, e0154888, Andres & Myers (2013) Earth Env. Sci. T. R. Soc. Edinburgh. 103, 383–398 and, Wang et al. (2017) Sci. Rep. 7, 42763, doi: 10.1038/srep42763.
Sampling strategy	The labial surface of the anterior most tooth, as close to the apex as possible, was sampled from the upper or lower jaw of pterosaur specimens (the jaw that was sampled depended on preservation quality). For the bats, the labial surface of a canine from the lower jaw, as close to the tooth apex as possible, was sampled. No preference was given to the right or left canine. Sample sizes for bats and pterosaurs were largely determined by museum availability and specimen preservation quality, e.g. only specimens with teeth preserved in situ in the jaws and with the original enamel preserved were sampled. However, previous dental microwear texture analyses have shown to be effective at discriminating diets between species or guilds even with low sample sizes of around 4-5 specimens per species/guild e.g. Purnell et al (2012) J. R. Soc. Interface. 9, 2225–2233 and Schultz et al. (2013) Wear 300, 169–179. The dietary guilds used for the bat and reptile dietary analyses are generally at least twice as large, thus low sample sizes for the present dietary analyses were not an issue. Some pterosaur genera that are only represented by one or two specimens limits the strength of the conclusions that can be drawn from these dietary reconstructions. This caveat is mentioned in the main and supporting texts. Other pterosaur genera that are represented by at least several specimens enabled more constrained conclusions to be drawn.
Data collection	JB collected the data. Moulds were taken of specimen teeth using a polyvinylsiloxane rubber at the museums where specimens were kept. These moulds were then filled with an Epoxy resin back in Leicester to create high quality cast replicas. Digital elevation models

	of tooth surfaces were captured and processed using the Alicona Infinite Focus Microscope with microwear data collected from these models using ISO areal textures parameters in Surfstand.
Timing and spatial scale	JB collected all moulds and microwear data over a 2 year period from 2016–2018. This included museum visits in the UK, Germany, Canada, Netherlands, China and USA and time spent on the Alicona microscope in Leicester. All museums from which specimens were sampled are included in the methods of the manuscript.
Data exclusions	The areal texture parameter Ssk (skewness of height distribution of surface) was excluded as data were log-transformed to suit the condition of normally distributed data for parametric testing. Ssk has negative values and thus could not be log-transformed. This left us with 21 texture parameters. For the pterosaur dietary evolution reconstructions, only the largest specimen of each genus was included to rule out any confounding effects of ontogeny. These exclusions are explained in the methods section of the manuscript.
Reproducibility	All attempts to repeat the experiment were successful. The reptile and bat dietary analyses were performed independently of each other. The pterosaur dietary evolution reconstructions using each of the three phylogenies were performed independently of each other.
Randomization	Randomisation was performed to an extent in that the reptiles and bats were independently assigned to dietary guilds based on the literature and our classification system (see Fig. S1) and so for analyses the species identity of the tooth textures did not need to be known, only the guild. Randomisation was not performed for pterosaurs as this study explicitly aimed to reconstruct their diets based on their tooth microwear texture characteristics. The identity of each datum therefore needed to be known during analysis.
Blinding	Blinding was performed to an extent in that the reptiles and bats were independently assigned to dietary guilds and so for analyses the species identity of the tooth textures did not need to be known, only the guild. Blinding was not performed for pterosaurs as this study explicitly aimed to reconstruct their diets based on their tooth microwear texture characteristics. The identity of each datum therefore needed to be known during analysis.

Did the study involve field work? ☐ Yes ☒ No

Reporting for specific materials, systems and methods

We require information from authors about some types of materials, experimental systems and methods used in many studies. Here, indicate whether each material, system or method listed is relevant to your study. If you are not sure if a list item applies to your research, read the appropriate section before selecting a response.

Materials & experimental systems

n/a	Involved in the study
☒	☐ Antibodies
☒	☐ Eukaryotic cell lines
☐	☒ Palaeontology and archaeology
☒	☐ Animals and other organisms
☒	☐ Human research participants
☒	☐ Clinical data
☒	☐ Dual use research of concern

Methods

n/a	Involved in the study
☒	☐ ChIP-seq
☒	☐ Flow cytometry
☒	☐ MRI-based neuroimaging

Palaeontology and Archaeology

Specimen provenance	No fieldwork was conducted for this study. All pterosaur specimens were sampled from publicly accessible museum collections. The provenance data for all pterosaur specimens, according to museum records, are found in Supplementary Data 1 (https://doi.org/10.5281/zenodo.4018876).
Specimen deposition	Pterosaur specimens are located in the following publicly accessible museums: Bayerische Staatsammlung für Paläontologie und Geologie, Munich, Germany (BSPG); British Geological Survey, British Geology Collections, Keyworth, UK (GSM); Institute of Vertebrate Paleontology and Paleoanthropology, Beijing, China (IVPP); Museum für Naturkunde der Humboldt-Universität Berlin, Berlin, Germany (MB); Natural History Museum, London, UK (NHMUK); Paleontological Museum of Liaoning, Shenyang, China (PMOL); Staatliches Museum für Naturkunde, Karlsruhe, Germany (SMNK); Staatliches Museum für Naturkunde, Stuttgart, Germany (SMNS); Teylers Museum, Haarlem, Netherlands (TM); Royal Tyrrell Museum of Palaeontology, Drumheller, Canada (TMP). This institution list is provided in the methods section of the manuscript and the full specimen list and museum record information is provided in Supplementary Data 1 (https://doi.org/10.5281/zenodo.4018876).
Dating methods	No new dates were provided for the pterosaur specimens. All stratigraphic dates were obtained from the literature or the Palaeobiology Database. This is explained in the manuscript and the dates are provided in Supplementary Data 1 (https://doi.org/10.5281/zenodo.4018876).

☒ Tick this box to confirm that the raw and calibrated dates are available in the paper or in Supplementary Information.

Ethics oversight	No ethical approval was needed for this study as all sampled pterosaurs are available in publicly accessible museum collections. The study protocol to sample the pterosaurs was approved by the same museums mentioned in the specimen deposition box above. This information is provided in the methods section of the manuscript.

Note that full information on the approval of the study protocol must also be provided in the manuscript.
