# Supplementary material for: Dietary diversity and evolution of the earliest flying vertebrates revealed by dental microwear texture analysis
Source: Nat Commun. 2020 Oct 28;11:5293. doi: 10.1038/s41467-020-19022-2 (PMC7595196; doi:10.1038/s41467-020-19022-2)
Supplement: Supplementary file 4 — Description of Additional Supplementary Files [file 41467_2020_19022_MOESM4_ESM.pdf]

Description of Additional Supplementary Files

File Name: Supplementary Data 1
Description: Specimens and raw data generated and used for dental microwear texture analysis. a, extant reptile and bat specimens and; b, pterosaur specimens. Includes raw data from the 22 ISO texture parameters used in analysis N.B. These data were logtransformed after normality testing and Ssk were thus excluded from further analysis as negative values cannot be log-transformed. Includes PC 1 and PC 2 scores from the reptile and bat texture-dietary spaces and the lower jaw lengths (mm) of all sampled specimens.

File Name: Supplementary Data 2
Description: Extant reptile (crocodilians and varanid lizards) and bat species, sample sizes and dietary guild assignments based on stomach and/or faecal content studies that give quantitative dietary breakdowns (%). Constituents can be measured as a percentage of total volume of samples or as a percentage of the frequency in which prey items appear in specimen stomachs/faecal content.

File Name: Supplementary Data 3
Description: ANOVA results (4 d.p) of ISO texture parameters between a, reptile and; b, bat dietary guilds. Data log transformed and scale limited using 5th order polynomial and robust Gaussian filter. Texture parameters exhibiting significant differences after application of the Benjamini-Hochberg procedure shown in bold. All ANOVAs were two-tailed tests and were performed independently of each other.

File Name: Supplementary Data 4
Description: International Organisation for Standardisation (ISO) 3D texture parameter definitions and categorisations. Many parameters are derived from the areal material ratio curve; a cumulative probability density function derived from the scale-limited tooth surface by plotting the cumulative percentage of the tooth surface against height The peaks, valleys and core material of tooth surfaces are defined on the basis of this curve, with the core for material ratio parameters equivalent to the volume that lies between the heights of the surface delimited by the extrapolated intercept of the minimum slope of the curve. Core, peaks and valleys for volume parameters are defined using slightly different thresholds. Note that Sds and Ssc predate ISO 25178.

File Name: Supplementary Data 5
Description: Dietary correlations for a, reptile and; b, bat dietary guilds. Spearman's rank correlations of PC axes 1 and 2 against dietary characteristics. Significant correlations after application of the Benjamini-Hochberg procedure shown in bold. The false discovery rate for the procedure was set at 0.05. All correlations were two-tailed tests and were performed independently of each other.

File Name: Supplementary Data 6
Description: Estimated ancestral PC 1 and 2 values for each node from the three phylogenies used in ancestral pterosaur dietary state reconstructions. (a) Lü et al. 1; (b) Andres & Myers 2; (c) Wang et al. 3. The variance and the upper and lower 95% confidence internal values are given for each node. All values to 4 d.p.

File Name: Supplementary Code 1

Description: Example R code used for the pterosaur dietary evolution reconstructions. Analyses were conducted for three separate phylogenetic hypotheses. Example code provided for the Andres phylogenetic hypothesis.
